# Dietary fat quantity and composition influence hepatic lipid metabolism and metabolic disease risk in humans

**DOI:** 10.1242/dmm.050878

**Published:** 2025-01-29

**Authors:** Nikola Srnic, Felix Westcott, Eleanor Caney, Leanne Hodson

**Affiliations:** ^1^ Oxford Centre for Diabetes, Endocrinology and Metabolism, University of Oxford, Oxford OX3 7LE, UK; ^2^ Oxford NIHR Biomedical Research Centre, Churchill Hospital, Oxford OX3 7LE, UK

**Keywords:** Dietary fat, Fat metabolism, Hepatocyte, Lipid droplet, Liver

## Abstract

The excessive accumulation of intrahepatic triglyceride (IHTG) in the liver is a risk factor for metabolic diseases, including type 2 diabetes and cardiovascular disease. IHTG can excessively accumulate owing to imbalances in the delivery, synthesis, storage and disposal of fat to, in and from the liver. Although obesity is strongly associated with IHTG accumulation, emerging evidence suggests that the composition of dietary fat, in addition to its quantity, plays a role in mediating IHTG accumulation. Evidence from human cross-sectional and interventional studies indicates that diets enriched with saturated fat compared to other fat types and carbohydrates produce divergent effects on IHTG content. However, the mechanistic reasons for these observations remain unknown. Given the challenges of investigating such mechanisms in humans, cellular models are needed that can recapitulate human hepatocyte fatty acid metabolism. Here, we review what is known from human studies about how dietary fat, its quantity and composition contribute to IHTG accumulation. We also explore the effects of fatty acid composition on hepatocellular fat metabolism from data generated in cellular models to help explain the divergences observed in *in vivo* studies.

## Teaser

**Summary:** In this Review, we discuss different ways of accumulating intrahepatic triglyceride and the influence that dietary fat composition and quantity have on metabolic disease risk.

## Introduction

The liver has a pivotal role in regulating whole-body lipid and glucose metabolism. In the transition from the fasted to fed state, there is an increased flux of nutrients to the liver, to which the liver responds via a series of complex metabolic cascades, to rapidly transition between the tasks of energy storage and supply. Although the liver is a flexible organ in metabolic terms, chronic perturbations in this organ's metabolic pathways, such as in lipid handling, can impact an individual's metabolic health. For example, an increased retention of fat within the liver [known as intrahepatic triglyceride (IHTG; see Glossary, Box 1)], which often co-exists with obesity, is strongly associated with an increased risk of insulin resistance (Box 1), type 2 diabetes (T2D; Box 1) and cardiovascular disease (CVD) (Gaggini et al., 2013; Powell-Wiley et al., 2021). Excessive IHTG content [known as steatosis (Box 1)] is a hallmark of fatty liver disease, which, until recently, was known as non-alcoholic fatty liver disease (NAFLD; Box 1), but is now called metabolic dysfunction-associated steatotic liver disease (MASLD; Box 1) (Rinella et al., 2023). Both NAFLD and MASLD can progress to inflammation, fibrosis and, ultimately, cirrhosis (Fabbrini and Magkos, 2015; Rinella et al., 2023). Although the terminology has changed from NAFLD to MASLD (Rinella et al., 2023), we will refer to the terminology/definition used by the authors of the respective studies we refer to throughout our Review.

**Fig. 1.**
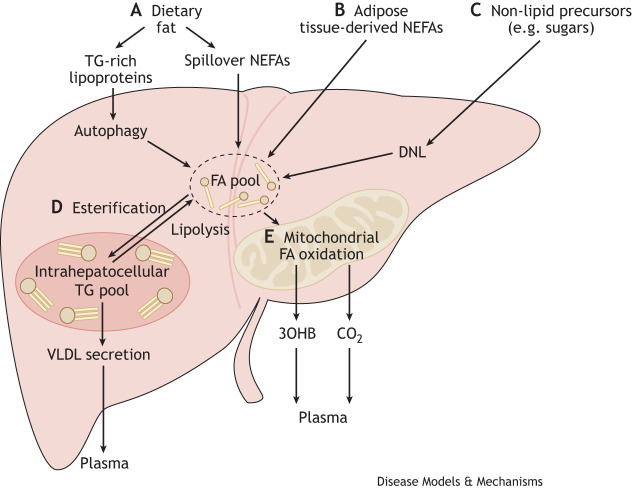
**Overview of human hepatic fatty acid metabolism.** Dietary fat can be directly taken up into hepatocytes in the human liver. (A,B) It reaches the liver via two main routes: as dietary fat, delivered in the form of triglyceride (TG)-rich lipoproteins or spillover from chylomicron–TG lipolysis in the form of non-esterified fatty acids (NEFAs) (A), and as a result of adipose tissue lipolysis (B). TG-rich lipoproteins are degraded via autophagic pathways, which liberate fatty acids (FAs) that are incorporated into the intrahepatocellular FA pool. (C) Within hepatocytes, FAs can be synthesized from non-lipid precursors through *de novo* lipogenesis (DNL). (D,E) From the intracellular FA pool, FAs can be esterified and stored as triglyceride in lipid droplets, then secreted as TG within very-low-density lipoproteins (VLDLs) (D), or enter oxidation pathways in mitochondria to form ketones, such as 3-hydroxybutyrate (3OHB), or carbon dioxide (CO_2_) (E).

**Fig. 2.**
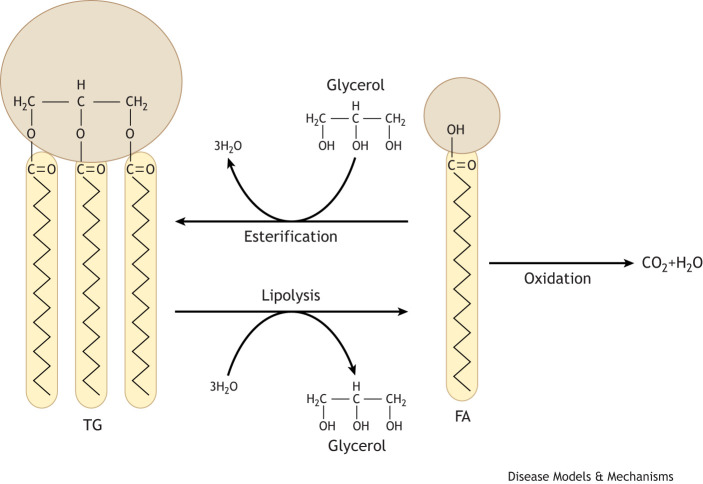
**FA esterification, lipolysis and oxidation.** Three FA molecules can combine with glycerol to be esterified into a TG, and this reaction is reversed during lipolysis. FAs can also be oxidized in the mitochondria to release CO_2_ and water (H_2_O).

A human liver's triglyceride (TG; Box 1) content is determined by a complex balance that is established between the amount and potentially type of fat that enters the liver (from adipose tissue and from dietary sources), the amount that is synthesized in the liver from non-lipid precursors (e.g. sugars and amino acids) via the *de novo* lipogenesis (DNL; Box 1) pathway, and the amount that is removed from the liver via oxidation (Box 1) [the TCA cycle and ketogenesis (Box 1)] and secretion pathways (Fig. 1). Within the liver, endogenous and exogenous sources of fatty acids (FAs) mix; most FAs are broadly partitioned between two pathways – oxidation and esterification (Box 1) (Fig. 2). They then form predominantly, but not exclusively, TG, which can either be stored in lipid droplets (LDs) (Box 1) or secreted as very-low-density lipoproteins [VLDLs (Box 1)] into the systemic circulation (Hodson and Gunn, 2019). Insulin plays an instrumental role in regulating FA fluxes to, and partitioning within, the liver, as it decreases FA flux from the adipose tissue to the liver and shifts hepatocellular metabolism away from oxidation toward esterification (McGarry et al., 1973; Lewis et al., 2002). The liver can also store TG to accommodate FAs that have accumulated in excess of requirements for oxidation and/or secretion in VLDLs (Hodson and Gunn, 2019).

The amount of dietary fat entering the liver depends on several factors, including the quantity and frequency with which it is consumed, along with an individual's phenotype. Dietary fat enters the liver as chylomicron (Box 1)-derived spillover FAs (Box 1) or as chylomicron remnants (Box 1) (Fig. 1) (Hodson and Gunn, 2019). Although it has been consistently demonstrated that overconsuming dietary fat, as excess calories, increases IHTG content (Hodson et al., 2020; Yki-Järvinen et al., 2021), evidence is emerging to suggest that composition of dietary fat might also influence IHTG accumulation (Hodson et al., 2020; Yki-Järvinen et al., 2021).

Here, we explore what is known about the effects of dietary fat composition (Box 2) on hepatic fat metabolism and how dietary fat composition might influence IHTG accumulation, and subsequently how dietary fat quantity and composition can influence metabolic disease risk in humans. Although it is acknowledged that the use of preclinical animal models has the potential to elucidate the further underlying mechanisms responsible for the complex cascade of events that may lead to IHTG accumulation, there is limited work focusing on exclusively dietary fat composition. Therefore, here we review available evidence from human *in vivo* studies, from cellular *in vitro* studies and, where relevant, from animal studies that offer mechanistic insight.

**Box 1. Glossary**

**Autophagy:** a process by which a cell breaks down and destroys old, damaged or abnormal proteins and other substances in its cytoplasm (the fluid inside a cell). The breakdown products are then recycled for important cell functions, especially during periods of stress or starvation.

**Bioactive lipid species:** molecules that form in response to, or act on, specific stimuli, affecting cell function, often signalling and regulating cellular functions, including metabolism.

**Ceramides:** a family of waxy lipid molecules. A ceramide is composed of sphingosine and a fatty acid joined by an amide bond.

**Chylomicrons:** large triglyceride-rich particles (lipoprotein) made by the intestine after the ingestion of dietary fat, which are involved in the transport of dietary triglycerides and cholesterol to peripheral tissues and liver.

**Chylomicron-derived spillover fatty acids:** in the process of hydrolysing fat from chylomicrons, lipoprotein lipase breaks down chylomicron–triglyceride into fatty acids, which are then transported into cells. Some of these fatty acids that are liberated from chylomicrons–triglyceride escape cellular uptake and are carried to other organs like the liver; these are known as chylomicron-derived spillover fatty acids.

**Chylomicron remnants:** particles that are formed after adipose and skeletal muscle tissue hydrolysis of triglyceride from chylomicron particles.

**Coronary heart disease (CHD):** characterized by the narrowing or blocking of the heart's blood vessels (coronary blood vessels) due to high cholesterol and plasma lipids causing fatty plaque buildup. This restricts blood flow to the heart muscle, leading to chest pain or a heart attack (myocardial infarction).

**Cross-sectional study:** observational study that examines a cohort or population at a single point in time

**Diacylglycerol (DAG):** a fat molecule that is composed of two fatty acids, which are each joined to a glycerol molecule by an ester bond. A fatty acid can be added to DAGs to form triglycerides, a fatty acid can be liberated from DAG for energy generation, and DAGs themselves function as bioactive lipids (see above).

***De novo* lipogenesis (DNL):** a metabolic pathway that makes fatty acids from non-lipid precursors such as sugars or amino acids. In humans, it primarily occurs within hepatocytes.

**Esterification:** a chemical reaction that joins an acid (e.g. a fatty acid) with an alcohol (e.g. glycerol) to form an ester. This is the opposite process to lipolysis.

**Extrahepatic tissue fat metabolism:** the storage, breakdown and handling of fat molecules in tissues that are not the liver.

**Glycaemic index (GI):** a measure used to describe how much a certain food increases blood sugar levels after consumption. A higher GI food (e.g. honey or white sugar) will increase blood sugars more rapidly than a lower GI food (e.g. beans or whole grains).

**Hepatocytes:** the main cell type in the liver responsible for carrying out most of the metabolic, secretory and detoxifying functions of the liver.

**HepG2 cells:** a human male liver cancer cell line typically used to model human hepatocytes *in vitro*.

**High-fat low-carbohydrate (HFLC) diet:** a diet that is typically 50-60% total energy from fat and 20-30% total energy from carbohydrate.

**Huh7 cells:** a human male liver cancer cell line typically used to model human hepatocytes *in vitro*.

**Hypercaloric diet:** a diet in which a person's energy consumption exceeds their energy expenditure.

**Hypocaloric diet:** a diet in which a person's energy consumption is less than their energy expenditure.

**Insulin resistance:** a state in which a cell, tissue, organ or body does not respond to and trigger the metabolic cascades normally activated by insulin.

**Intrahepatic triglyceride (IHTG):** triglyceride (see below) that is stored within liver cells.

**Intrahepatocellular:** occurring within hepatocytes, the major cell type of the liver.

**Interventional study:** a research study that investigates how a certain population or cohort responds to a certain intervention (drug, device, diet, activity or procedure). In some instances, these studies are referred to as clinical trials.

**Isocaloric diet:** a diet in which a person's energy consumption matches their energy expenditure.

**Ketogenesis:** a fat oxidation pathway, primarily in the liver in humans, whereby fatty acids are incompletely broken down to form ketone bodies and secreted into the blood so that the ketone bodies can be broken down for energy generation by tissues that are unable to break down fatty acids.

**Lipid droplet (LD):** an intracellular organelle specialized for storing fat; it is composed of a fat core and protein surface.

**Lipophagy:** a type of autophagy (see above) that breaks down lipid droplets and fat.

**Lipotoxicity:** cell damage that occurs in response to lipid overload.

**Lipolysis:** the process by which fatty acids are liberated from triglycerides. This is the opposite process to esterification.

**Low-fat high-carbohydrate (LFHC) diet:** a diet that is typically 20-30% total energy from fat and 60-70% total energy from carbohydrate.

**Metabolic dysfunction-associated steatotic liver disease (MASLD):** a chronic liver disease [previously termed non-alcoholic fatty liver disease (NAFLD)] that is characterized by excessive fat accumulation within the liver in the presence of at least one risk factor for metabolic syndrome.

**Metabolic non-alcoholic fatty liver disease (NAFLD):** a chronic liver disease [recently redefined as metabolic dysfunction-associated steatotic liver disease (MASLD)] that is characterized by excessive fat accumulation within the liver. Individuals with metabolic NAFLD have multiple features of metabolic syndrome and are not carriers of the common I148M variant in patatin-like phospholipase domain-containing protein 3 (*PNPLA3*) gene.

**Monounsaturated fatty acid (MUFA):** a fatty acid with only one carbon–carbon bond in the carbon chain (see Box 2).

**Non-alcoholic steatohepatitis (NASH):** a more-severe form of NAFLD (see below) characterized by excessive fat accumulation in the liver as well as inflammation.

**Non-alcoholic fatty liver disease (NAFLD):** a chronic liver disease [recently redefined as metabolic dysfunction-associated steatotic liver disease (MASLD)] that is characterized by excessive fat accumulation within the liver and minimal inflammation, and can progress to more-severe liver disease with inflammation to very-severe liver disease, like cirrhosis.

**Oxidation:** a chemical reaction whereby electrons are extracted from an atom. In the context of lipid metabolism, it refers to the process of extracting electrons from energy substrates, such as lipids, to power the formation of high-energy phosphates, such as adenosine triphosphate (ATP), for other cellular processes.

**Perilipin (PLIN) proteins:** proteins found on the outside of lipid droplets that form the border between the lipid droplet core and cellular cytoplasm. These proteins are involved in fat metabolism, mobilization and storage.

**PNPLA3 non-alcoholic fatty liver disease (NAFLD):** a chronic liver disease [recently redefined as metabolic dysfunction-associated steatotic liver disease (MASLD)] that is characterized by excessive fat accumulation within the liver. Individuals with PNPLA3 NAFLD have minimal features of metabolic syndrome and are carriers of the common I148M variant in patatin-like phospholipase domain-containing protein 3 (*PNPLA3*) gene.

**Polyunsaturated fatty acid (PUFA):** a fatty acid with two or more carbon–carbon double bonds in the carbon chain (see Box 2).

**Primary human hepatocytes (PHHs):** human liver cells that are directly isolated from human donors for *in vitro* cell culture.

**Reactive oxygen species (ROS):** highly reactive molecules that can cause cellular damage and are formed as byproducts from oxidation of energy substrates like fats and carbohydrates.

**Saturated fatty acid (SFA):** a fatty acid with no double bonds in the carbon chain (see Box 2).

**Stable-isotope tracer methodologies:** a research technique whereby a molecule of interest is labelled with a non-radioactive atom (i.e. a stable isotope) so that the molecule and its metabolites can be detected and followed as it is metabolized within a cell, organ or the body.

**Steatosis:** the pathological buildup of fat within a cell, tissue or organ.

**Sterol regulatory element-binding protein 1 (SREBP-1):** a transcription factor that regulates the expression of genes involved in lipid and cholesterol biosynthesis.

**Total energy (TE):** the total energy consumed in food and drink by an individual.

**Triglyceride (TG):** a type of fat molecule that is composed of three fatty acids, which are each joined to a glycerol molecule by an ester bond. Fatty acids are typically converted to triglycerides for storage within cells or transport throughout the body on triglyceride-rich lipoproteins.

**Triglyceride-rich lipoproteins (TRLs):** fat particles in the blood, for example very-low-density lipoproteins and chylomicrons, that have a lipid-rich core predominantly composed of triglyceride.

**Type 2 diabetes (T2D):** a common metabolic disease characterized by an inability to maintain normal blood sugar levels.

**Unsaturated fatty acid:** a fatty acid with at least one carbon–carbon double bond (see Box 2). It includes polyunsaturated and monounsaturated fatty acids.

**Very-low-density lipoprotein (VLDL):** a fat particle present in the blood that is secreted from the liver and transports fats from the liver to peripheral tissues such as the adipose, skeletal muscle and heart.

**Visceral adipose tissue:** fat tissue (adipocytes/adipose tissue) in the abdominal cavity around organs like the liver, pancreas and intestines.

**Box 2. Major types of dietary fatty acids**

Fat, a component of the diet, is predominantly consumed as triglycerides, with a minor contribution from cholesterol, phospholipids and fatty acids. To be absorbed, triglyceride needs to be broken down into three fatty acids and a glycerol molecule, whereas fatty acids and cholesterol can be directly absorbed without initial processing. Once absorbed by the small intestine, dietary fats are packaged into fat transport particles called chylomicrons for delivery to tissues like the liver. The predominant dietary fatty acids consumed are (1) saturated fatty acids (SFAs), (2) monounsaturated fatty acids (MUFAs) and (3) polyunsaturated fatty acids (PUFAs), and they can have different effects on a person's metabolic health, as briefly discussed below.

**SFAs**

SFAs are characterized by no double bonds in the carbon chain and contain the maximum number of hydrogens possible, and are therefore ‘saturated’ with hydrogen, as shown in the example below.

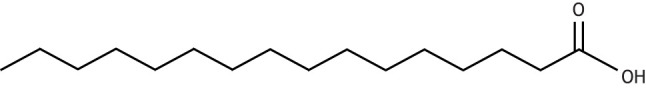

Dietary SFAs are found typically in fats from land animals (e.g. cream, butter, hard and mature cheese, meat fat), processed foods (e.g. pastries, cakes, biscuits, dark chocolate) and some seed oils (e.g. coconut, palm oils). Diets that contain high amounts of saturated fat have been associated with higher levels of plasma cholesterol, increased risk of obesity, metabolic syndrome, steatotic liver disease, type 2 diabetes and coronary heart disease; this list is not exhaustive.

**MUFAs**

MUFAs are characterized by one carbon–carbon double bond in their carbon chain. Most naturally occurring MUFAs in the *cis* configuration (carbon tails on either side of the double bond are on same side, as shown in the example below) as opposed to the *trans* configuration (carbon tails on either side of the double bond are on the opposite side).

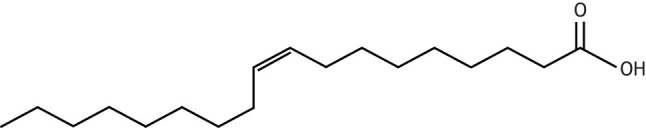

Dietary MUFAs are typically found in plant oils (e.g. olive oil, avocados, and certain nuts and seeds, such as almonds and peanuts), dairy and meat fats (e.g. meat fat, cheese, cream). Studies on diets enriched in monounsaturated fat are inconclusive, with some showing associations with lower rates of obesity, steatotic liver disease and cardiovascular disease, and other studies showing no difference.

**PUFAs**

PUFAs are characterized by two or more carbon–carbon double bonds in their carbon chain (see the example below). There are two groups of PUFAs: omega-6 and omega-3.

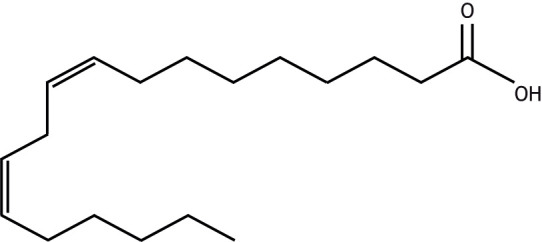

PUFAs are predominantly found in fats from plants (e.g. sunflower and sesame seed oils, walnuts, rapeseed oil, etc.) and marine foods (e.g. fish, fish oils). Diets enriched in PUFAs are often associated with lower levels of plasma cholesterol for omega-6, rates of obesity, steatotic liver disease and cardiovascular disease.

## Dietary patterns and metabolic disease risk in humans

The effect of dietary fat composition on metabolic disease risk was first described in the 1970s in the Seven Countries Study, which identified a positive association between saturated FA (SFA; Box 1) intake, total serum cholesterol and coronary heart disease (CHD; Box 1) risk (Keys, 1970). Although the relationship between cholesterol, SFA and CHD has been well described and thoroughly reviewed (Ference et al., 2017; Grundy et al., 2019; Griffin and Lovegrove, 2024), the relationship between dietary fat composition and the risk of developing other metabolic diseases, such as MASLD, remains unclear.

Most population-based observational studies have found that the consumption of excess calories or diets that are enriched in SFA, free sugars and/or processed foods are associated with greater rates of obesity, increased circulating plasma-TG levels, hepatic steatosis and insulin resistance (Trovato et al., 2016; Ferolla et al., 2013; Oddy et al., 2013). Findings from a limited number of cross-sectional studies (Box 1) that assessed dietary FA composition in individuals with and without NAFLD indicated that individuals with NAFLD and non-alcoholic steatohepatitis (NASH; Box 1) consumed more total fat, as well as more SFA and cholesterol, and less monounsaturated FA (MUFA; Box 1) and polyunsaturated FA (PUFA; Box 1), than did age- and body mass index (BMI)-matched individuals without NAFLD (Allard et al., 2008; Ferolla et al., 2013; Musso et al., 2003). In individuals with diagnosed NAFLD, this dietary pattern was associated with a higher degree of insulin resistance and with a higher likelihood of hepatic steatosis progressing to NASH, compared to that in individuals with NAFLD who had consumed a diet lower in SFA and higher in unsaturated FA (Box 1) (Aller et al., 2015; Chan et al., 2015; Kontogianni et al., 2014). Although self-reported food diaries and questionnaires can be prone to reporter bias (Hill and Davies, 2001), these findings are in line with more objective measures, such as the measurement of FA composition of plasma and intrahepatic TG (including lipidomic analysis of liver biopsies), which have found that patients with NAFLD and NASH have increased SFA and decreased unsaturated FA in their circulating plasma-TG and intrahepatic lipid pools, compared to individuals without NAFLD and NASH (Araya et al., 2004; Erickson et al., 2019; Puri et al., 2007, 2009; Sanders et al., 2018).

Although the consumption of dietary SFA and of unsaturated FA differs between patients with and without NAFLD, it has also been reported that those with NAFLD consume more free sugars and sugar-sweetened beverages than individuals without NAFLD (Chen et al., 2019; Ouyang et al., 2008; Porto et al., 2022; Tseng et al., 2023), indicating that dietary sugars might also contribute to the development of NAFLD. As sugars are a substrate for hepatic DNL, a pathway that primarily produces the SFA palmitate (C16:0), excess sugar consumption could result in a greater abundance of palmitate within hepatocytes (Box 1). In a study that assessed hepatic DNL in individuals with and without NAFLD, T2D or glycogen storage disease type 1a, Roumans et al. (2020) used ^1^H-magnetic resonance spectroscopy to demonstrate that although hepatic DNL did not correlate with IHTG content, it positively correlated with the hepatic SFA fraction and inversely associated with the MUFA, rather than with the PUFA, fraction (Roumans et al., 2020). These observations suggest that hepatic DNL can alter the FA composition, but not necessarily the total content of IHTG. Roumans and colleagues also found that the total IHTG content and liver PUFA fraction did not associate with hepatic insulin sensitivity, whereas the SFA fraction had a strong, negative correlation with, and the MUFA fraction had a significant positive association with, hepatic insulin sensitivity (Roumans et al., 2020). On the basis of these observations, the authors proposed that the amount of SFA in the liver negatively contributes to hepatic insulin sensitivity, rather than the total amount of fat, and that high rates of hepatic DNL play a role in altering hepatic FA composition (Roumans et al., 2020). However, palmitate can undergo elongation to stearate (C18:0) and desaturation by stearoyl-CoA desaturase (SCD; also known as SCD1) to the MUFAs palmitoleate (C16:1 n-7) and oleate (C18:1 n-9) (Hodson and Fielding, 2013). It is plausible, therefore, that if an individual has high rates of hepatic DNL but can desaturate newly made palmitate to either palmitoleate and/or oleate, then the effects of these high hepatic DNL rates on hepatic insulin sensitivity might be partly attenuated (and might thus attenuate MASLD development and progression as well). Alternatively, if individuals with high rates of hepatic DNL consume a high-SFA diet, it potentially can increase the amount of SFA within the liver. This increase may exceed the capacity of hepatic enzymes to desaturate and elongate palmitate, and lead to an accumulation of intrahepatic SFA, which may potentiate the development and progression of insulin resistance and MASLD. Thus, although observational studies have identified broad dietary patterns that are associated with liver steatosis, it is difficult to determine whether specific dietary FAs influence IHTG accumulation and/or NAFLD progression.

## Interventional studies of dietary fat

A limited number of interventional studies (Box 1) have investigated how dietary fat quantity and composition influence IHTG accumulation in cohorts of varying age, BMI and disease status, and are summarized in Table 1. To investigate the effects of dietary fat quantity on IHTG accumulation, participants consume diets with either fewer calories [hypocaloric (Box 1)], more calories [hypercaloric (Box 1)] or an equal number of calories [isocaloric (Box 1)] for their individual metabolic needs. These diets may also vary in macronutrient composition; for example, some participants may consume high-fat low-carbohydrate (HFLC) diets (Box 1), while others consume low-fat high-carbohydrate (LFHC) diets (Box 1). To study the effect of dietary fat composition on IHTG accumulation, participants typically consume the same amount of fat, but it may vary in composition; for example, some participants may consume a diet enriched in SFA and others a diet enriched in PUFA. There is currently no universally accepted definition for what constitutes a HFLC or LFHC diet, but it has been suggested that a HFLC diet is between 50% and 60% total energy (TE; Box 1) fat and 20-30% TE carbohydrate, and a LFHC diet is 20-30% TE fat and 60-70% TE carbohydrate (Noakes and Windt, 2017; Nordmann et al., 2006). For the purposes of this Review, we refer to the type of diet consumed (e.g. HFLC, LCHF, SFA-enriched, etc.) using the same terminology as that used by the authors of the discussed study.

**Table 1.** Interventional human studies investigating the effects of dietary fat composition on intrahepatic triglyceride content

Study	Participants	Diet	Intervention	Duration	Change in liver fat
Browning et al., 2011	18 M+F NAFLD 45±12 years 35±7 kg/m^2^	Hypo	Low-CHO diet (<20 g/day) vs low-kcal diet (∼1200-1500 kcal/day)	14 days	Low-CHO: ↓from 22±13% to 10±7% Low-kcal: ↓from 19±10% to 14±7%
Haufe et al., 2011	102 M+F 30-60 years** 25-45 kg/m^2^**	Hypo	Low-CHO diet (<90 g/day) vs low-fat diet (<20% TE)	6 months	Low-CHO: ↓∼47% Low-fat: ↓∼42% NSD between groups
Kirk et al., 2009	22 M+F 43.6±2.5 years 36.5±0.8 kg/m^2^	Hypo	High-CHO diet (>180 g/day) vs low-CHO diet (<60 g/day)	∼11 weeks	Low-CHO: ↓38.0±4.5% High-CHO: ↓44.5±13.5% NSD between groups
Lewis et al., 2006	18 M+F 50 years^‡^ (34-57 years)^¶^ 44 kg/m^2‡^ (40-51 kg/m^2^)^¶^	Hypo	VLCD	6 weeks	↓∼43%
Otten et al., 2016	70 F 50-70 years** 25-40 kg/m^2^**	Hypo	Paleo diet (40% TE fat) vs low-fat diet (25-30% TE fat)	6 months to 2 years	Paleo: ↓64% 6 months, ↓50% 2 years Low-fat: ↓43% 6 months, ↓49% 2 years
Steven et al., 2016	30 M+F T2DM 25-80 years** 27-45 kg/m^2^**	Hypo	VLCD; participants separated based on FPG at follow up Responders: <7 mmol/l Non-responders: >7 mmol/l	8 weeks followed by 6 months’ weight maintenance	Responders: ↓12.8±2.7% to 2.2±0.2% Non-responders: ↓8.2±1.1% to 2.2±0.1% NSD between groups
Bian et al., 2014	43 M+F 51±3 years 30.6±1.4 kg/m^2^	Hypo	6 days low-CHO diet (−1000 kcal/day, <20 g CHO/day) vs −1000 kcal/week as 30% TE fat (10% SFA, 10% MUFA and 10% PUFA), 50% TE CHO until ∼5% weight loss achieved	6 days vs 7 months	6 days low-CHO diet: from 11.1±1.3% to 8.4±1.3% 7 months 5% weight loss: from 10.8±1.5% to 7.8±1.4% NSD between groups
Schutte et al., 2022	100 M+F 61 years^‡^ (42-70 years)^§^ 31.2±3.3* kg/m^2^	Hypo	25% ER as a high-quality diet vs low-quality diet vs no-ER habitual diet	12 weeks	ER high-quality diet: ↓55% ER low-quality diet: ↓46% Weight-neutral habitual diet: ↑24% NSD between weight loss conditions
Crabtree et al., 2021	37 M+F 35±3 years 30.9±0.7 kg/m^2^	Hypo	KD+ketone salt vs KD+placebo vs low-fat diet	6 weeks	KD+ketone salt: ↓42% KD+placebo: ↓32% Low-fat diet: ↓52% NSD between groups
Sobrecases et al., 2010	30 M 23.9±0.4 years 22.6±0.2 kg/m^2^	Hyper	StdD+Fru (3.5 g/kg/FFM), SFA (+30% TE), or StdD+Fru+SFA (3.5 g/kg/FFM+30% TE)	7 days	Fru: ↑∼16% SFA: ↑∼86% Fru+SFA: ↑∼133%
Luukkonen et al., 2018	38 M+F 48±2 years 31±1 kg/m^2^	Hyper	Extra 1000 kcal/day as SFA vs UnSFA vs simple sugars	3 weeks	SFA: ↑∼55% UnSFA: ↑∼15% Sugar: ↑∼33%
Rosqvist et al., 2014	37 M+F 20-38 years** 18-27 kg/m^2^**	Hyper	Habitual diet+high-SFA or high-n-6 PUFA muffins	49 days	SFA: ↑0.56±1% PUFA: ↑0.04±0.24%
Rosqvist et al., 2019	60 M+F 42.3±9.5 years* 28.3±3.5* kg/m^2^**	Hyper	Habitual diet+high-SFA or high-n-6 PUFA muffins	8 weeks	SFA: ↑∼53% PUFA: ↓∼2%
Bjermo et al., 2012	61 M+F 30-65 years** 25-40 kg/m^2^**	Iso	n-6 PUFA enriched (15% TE) vs SFA enriched (15% TE)	10 weeks	n-6 PUFA: ↓0.9% SFA: ↑0.3%
Marina et al., 2014	13 M+F 18-55 years** >27 kg/m^2^**	Iso	Low-fat diet (20% TE) vs high-fat diet (55% TE)	4 weeks	Low-fat: ↓13.9±10.2% High-fat: NSD
Utzschneider et al., 2013	35 M+F 69.3±1.6 years 26.9±0.8 kg/m^2^	Iso	High-SFA diet (24% TE) vs low-SFA diet (7% TE)	4 weeks	High-SFA: NSD Low-SFA: ↓19.8±6%.
van Herpen et al., 2011	20 M 50-60 years** >27 kg/m^2^**	Iso	Low-fat diet (20% TE) vs high-fat diet (55% TE)^‡^	4 weeks	Low-fat: ↓∼13% High-fat: ↑∼17%
Westerbacka et al., 2005	10 F 43±5 years 33±4 kg/m^2^	Iso	Low-fat diet (16% TE) vs high-fat diet (56% TE)	14 days	Low-fat: ↓20±9% High-fat: ↑35±21%
Parry et al., 2020	16 M 47.9±1.1 years 27.7±0.4 kg/m^2^	Iso	High-SFA diet (45% TE fat, 20% TE SFA) vs high-sugar diet (20% TE fat, 20% TE sugar)	4 weeks	SFA: ↑∼39.0±10.0% Sugar: NSD
Basset-Sagarminaga et al., 2023	13 M+F 67±6* years 29.5±1.9* kg/m^2^	Iso	High-GI/SFA diet vs low-GI/SFA diet	2 weeks	Post high-GI/SFA diet: 3.3±0.6% Post low-GI/SFA diet: 2.4±0.5%

Participant data presented as mean±s.e.m. unless otherwise stated. ‘*’, s.d.; ‘^‡^’, median; ‘^§^’, range; ‘^¶^’, interquartile range; ‘**’, inclusion criteria or estimated range within which all participants are included; ‘↓’, decrease in liver fat; ‘↑’, increase in liver fat.

CHO, carbohydrate; ER, energy restricted; FFM, fat-free mass; FPG, fasting plasma glucose; F, female; Fru, fructose; GI, glycaemic index; Hyper, hypercaloric; Hypo, hypocaloric; Iso, isocaloric; KD, ketogenic diet; M, male; NAFLD, non-alcoholic fatty liver disease; NSD, no significant difference; Paleo, paleolithic; PUFA, polyunsaturated fatty acids; SFA, saturated fatty acids; StdD, standard diet; TE, total energy; T2DM, type 2 diabetes mellitus; UnSFA, unsaturated fatty acids; VLCD, very-low-calorie diet.

### Findings from hypocaloric studies

A consistent finding from hypocaloric studies is that regardless of macronutrient (e.g. fat, carbohydrate) or specific dietary fat composition, decreased caloric intake reduces body weight and IHTG content (Browning et al., 2011; Kirk et al., 2009; Crabtree et al., 2021; Lewis et al., 2006; Otten et al., 2016; Steven et al., 2016) (Table 1). This decrease in IHTG accumulation has been consistently observed in people who are overweight, obese and/or have NAFLD or T2D, and who have undertaken hypocaloric interventions from 14 days for up to 2 years (Table 1), as previously reviewed (Parry and Hodson, 2017). In five studies that compared the effects on IHTG content of hypocaloric HFLC or LFHC diets, most reported similar reductions in body weight and IHTG across the two diet types and irrespective of dietary composition (Browning et al., 2011; Haufe et al., 2011; Kirk et al., 2009; Bian et al., 2014; Schutte et al., 2022; Otten et al., 2016). Recently, Schutte et al. (2022) explored the effects of two 25% energy-restricted LFHC diets on weight loss and IHTG content in 110 participants with obesity aged 40-70 years. One diet was enriched in unsaturated FA, fibre and plant protein (high nutrient quality), whereas the other was enriched in SFA and free sugars (low nutrient quality), and both were consumed for 12 weeks. This study found that there was no difference in the reduction in IHTG content between the two diet types. This is despite the fact that the high-nutrient-quality energy-restricted diet induced a 2.1 kg greater weight loss (*P*=0.007) than did the low-nutrient-quality energy-restricted diet, which might be a result of the greater variation in caloric restriction between the groups (Schutte et al., 2022). These observations, along with other studies, indicate that dietary fat composition has minimal impact on changes in IHTG content, in the context of a hypocaloric diet.

### Findings from hypercaloric studies

The consumption of a hypercaloric fat-enriched diet appears to result in greater IHTG accumulation, relative to that of a hypercaloric carbohydrate-enriched diet, despite both diets inducing similar levels of weight gain (Luukkonen et al., 2018; Sobrecases et al., 2010). These findings suggest that, in conditions of caloric excess, dietary fat quantity and/or composition influences the extent of IHTG accumulation (Table 1). For example, Luukkonen et al. (2018) reported that when overweight participants consumed an extra 1000 kcal/day as either SFA, unsaturated FA or simple sugars for 3 weeks, IHTG content increased to a greater extent following the SFA diet, compared to the unsaturated FA or simple sugar diets. Moreover, it was observed that after 3 weeks of consuming the SFA diet, markers of insulin resistance and plasma ceramides (Box 1) increased; these markers remained unchanged in those consuming the unsaturated FA or simple sugar-enriched diets (Luukkonen et al., 2018). These findings indicate that increasing SFA content, in the context of a high-fat hypercaloric diet, promotes a greater increase in IHTG accumulation and an increased risk of metabolic disease. Overfeeding studies in lean and overweight participants, lasting between 1 and 8 weeks, have found that overfeeding with SFA, compared to overfeeding with unsaturated FA or fructose, leads to greater levels of IHTG accumulation, and to increased fasting plasma total cholesterol, low-density lipoprotein cholesterol and apolipoprotein-B concentrations (Rosqvist et al., 2014, 2019) (Table 1). Taken together, these hypercaloric diet studies indicate that the effects of weight gain on IHTG content are exacerbated by the consumption of SFA diets, relative to that of unsaturated FA or sugar-enriched diets.

### Findings from isocaloric studies

In overweight and obese men and women, with and without NAFLD, the consumption of an isocaloric LFHC diet, or that of a low-SFA diet, for between 2 and 10 weeks, decreased their IHTG content (Bjermo et al., 2012; Basset-Sagarminaga et al., 2023; van Herpen et al., 2011; Westerbacka et al., 2005; Utzschneider et al., 2013). By contrast, isocaloric HFLC or high-SFA diets increased or did not change the IHTG content of the study participants (Basset-Sagarminaga et al., 2023; Bjermo et al., 2012; Utzschneider et al., 2013; van Herpen et al., 2011; Westerbacka et al., 2005; Parry et al., 2020; Marina et al., 2014) (Table 1). To investigate the effects of specific dietary FAs on IHTG content, 61 overweight and obese participants consumed an isocaloric LFHC diet enriched with either SFA or PUFA for 10 weeks (Bjermo et al., 2012). Following the consumption of the PUFA-enriched diet, the participants' IHTG content decreased by 16%. By contrast, IHTG content increased by 34% in those consuming the SFA-enriched diet (Bjermo et al., 2012). Using a randomized crossover study design, Basset-Sagarminaga et al. (2023) compared the effects of a 2-week isocaloric low-glycaemic index (GI) (Box 1), low-SFA diet to those of a high-GI, high-SFA diet in 13 overweight and obese men and women. They found that IHTG accumulation was significantly lower after 2 weeks in those who had consumed the low-GI, low-SFA diet than in those who had consumed the high-GI, high-SFA diet; changes in IHTG content were not correlated with changes in body weight (Basset-Sagarminaga et al., 2023). Moreover, following the consumption of a test meal, the high-GI, high-SFA diet led to significantly greater postprandial TG and glucose concentrations than did the low-GI, low-SFA diet (Basset-Sagarminaga et al., 2023). Our research group has previously compared, in a randomized crossover trial, the metabolic effects of a 4-week isocaloric diet enriched in SFA to a diet enriched in free sugars in 16 overweight men (Parry et al., 2020). This study found that the isocaloric SFA-enriched diet increased IHTG content to a greater extent, and led to greater postprandial glucose and insulin excursions, than did the free sugar-enriched diet; changes in IHTG content did not correlate with changes in weight (Parry et al., 2020). Despite the differences in the design, duration, measured outcomes (e.g. plasma biochemical parameters) and participant populations among these hypercaloric and isocaloric intervention studies, they demonstrate the divergent effects of dietary SFA and of unsaturated FA on IHTG accumulation.

Although increased total liver fat content is a known risk factor for metabolic diseases, such as insulin resistance, T2D and CVD, the composition of liver fat might play an important role in mediating such disease risk. In support of this concept, Luukkonen et al. (2016) observed that the liver lipidome of patients with elevated IHTG content and multiple features of metabolic syndrome, and who were not carriers of the common I148M variant in patatin-like phospholipase domain-containing protein 3 (*PNPLA3*) gene (‘metabolic NAFLD’; Box 1) was markedly enriched in saturated and monounsaturated TG, as well as in free FAs, markers of *de novo* ceramide synthesis, and ceramides, compared to patients without metabolic NAFLD. In contrast, the liver lipidome of individuals with elevated IHTG content, minimal features of metabolic syndrome, and who were carriers of the common I148M variant in *PNPLA3* gene (‘PNPLA3 NAFLD’; Box 1) was enriched in polyunsaturated TG, and had other lipid types (e.g. ceramides) that were no different from those of individuals with ‘non-PNPLA3 NAFLD’ (Luukkonen et al., 2016). Finally, the metabolic NAFLD group showed characteristics associated with insulin resistance, whereas the PNPLA3 NAFLD group did not, despite having comparable total liver fat (Luukkonen et al., 2016). Other studies have reported that the composition of hepatic lipids from patients with NAFLD or NASH is enriched with SFA, MUFA and diacylglycerols (DAGs; Box 1), and is depleted of PUFA, compared to that of hepatic lipids from non-NAFLD obese controls (Araya et al., 2004; Puri et al., 2007). Thus, although it appears that IHTG FA composition can influence an individual's metabolic health, and that a SFA-enriched/PUFA-depleted FA profile is associated with adverse metabolic measures, the mechanisms underpinning these observations remain to be elucidated.

Taken together, the findings from dietary studies clearly demonstrate that dietary fat quantity can impact IHTG content, with emerging evidence showing that the FA composition of IHTG can also influence the accumulation of IHTG. It is plausible that the FA composition of the diet has the potential to influence the FA composition of IHTG, and, given the divergence in the reported effects of SFA and PUFA on IHTG accumulation, this would suggest that there are differences in intrahepatic metabolism of specific FAs.

## Investigating the differential handling of dietary FAs in humans

Several potential mechanisms have been proposed to explain the divergence in IHTG accumulation following the consumption of a SFA- compared to a PUFA-enriched diet. These mechanisms include that SFA (1) does not preferentially enter oxidation pathways relative to PUFA (DeLany et al., 2000; Parry et al., 2021); (2) causes the synthesis of biologically active lipids, such as ceramides or DAGs (Luukkonen et al., 2016, 2018); and (3) induces endoplasmic reticulum (ER) stress responses (Kumashiro et al., 2011).

In a small study (*n*=4 men), in which stable-isotope tracer methodologies (Box 1) were used, DeLany et al. (2000) observed that whole-body FA oxidation decreased with increasing FA-chain length and/or with decreased saturation of FAs. By using ^13^C-labelled FAs, our research group found that the oxidation of dietary lineolate (a PUFA) was greater than that of dietary palmitate (a SFA) in healthy individuals consuming their habitual diet (Parry et al., 2021). In support of these observations, in mitochondria isolated from rat livers, carnitine-palmitoyl transferase I (CPT1; also known as CPT1A), the enzyme that catalyses the rate-determining step of mitochondrial FA oxidation, had a higher binding affinity and transport rate for PUFA compared to SFA (Gavino and Gavino, 1991). Although it remains unclear whether human hepatic CPT1 has a higher binding affinity and transport rate for PUFA compared to SFA, it could be speculated, based on a limited number of observations (DeLany et al., 2000; Parry et al., 2021), that dietary PUFA, relative to SFA, is preferentially partitioned into mitochondrial oxidation pathways, which would reduce FA availability for TG synthesis and, thus, IHTG accumulation.

Most other proposed intrahepatic mechanisms to explain the greater IHTG accumulation with consumption of a SFA- compared to a PUFA-enriched diet, suggest that when there is an excess of intracellular SFA, it increases hepatocellular ceramides, DAGs and/or the resulting ER stress, which leads to cellular derangements and to IHTG accumulation (Luukkonen et al., 2016; Kumashiro et al., 2011; Magkos et al., 2012). Indeed, the consumption of a SFA-enriched diet in overweight and obese men and women is reported to increase plasma ceramides (Luukkonen et al., 2018; Rosqvist et al., 2019), and the livers of patients with NAFLD compared to those of individuals without NAFLD have a greater abundance of ceramides (Luukkonen et al., 2016) and DAGs (Kumashiro et al., 2011; Magkos et al., 2012; Puri et al., 2007), which are positively associated with hepatic steatosis, insulin resistance and inflammation (as reviewed by Petersen and Shulman, 2017). Determining the potential roles of these bioactive lipid species (Box 1), namely ceramides and DAGs, in IHTG accumulation in humans is challenging, as it requires dissecting and disentangling the temporal responses to excess SFA.

The effect of FA composition on extrahepatic tissue fat metabolism (Box 1) is also thought to influence IHTG accumulation by modulating FA flux to the liver. Elevated visceral adipose tissue (Box 1) lipolysis (Box 1) has been suggested to increase IHTG accumulation (Boden, 1997). However, there is sparse evidence from *in vivo* human studies that a SFA-enriched diet produces divergent effects on adipose tissue lipolysis compared to a PUFA-enriched diet (Luukkonen et al., 2018). However, our group has previously shown that FA trafficking in humans is decreased in obese compared to lean adipose tissue (McQuaid et al., 2011), and that this might lead to the increased delivery of dietary fat to the liver as remnant TG-rich lipoproteins (TRLs; Box 1); evidence for an effect of dietary fat composition on this pathway has yet to be demonstrated. Finally, the secretion of TG from the liver as VLDL is an important disposal route for IHTG; however, evidence on how dietary fat composition might influence this is lacking. To elucidate the mechanisms that underpin the differential intrahepatocellular (Box 1) handling of SFA and PUFA, cellular *in vitro* models that capture the complexity of *in vivo* human FA metabolism are needed, as we discuss next in this Review.

## Investigating hepatic fat metabolism in cellular models

Compared to other metabolic substrates, FAs are potentially the primary driver of intrahepatocellular lipid accumulation *in vitro* and are frequently included in cellular models to induce hepatocellular lipid accumulation (Green et al., 2015b). Studying FA metabolism using *in vitro* cellular models is typically undertaken using two-dimensional cultures of a single cell line (monocultures). However, there is increasing work probing hepatocellular FA metabolism using two-dimensional co-cultures of hepatocytes with other intrahepatic cell types (such as immune cells) and using three-dimensional culturing techniques. Various cell lines, methods and developments in hepatocellular cell culture have been comprehensively reviewed (Pelechá et al., 2021; Panchuk et al., 2024).

**Fig. 3.**
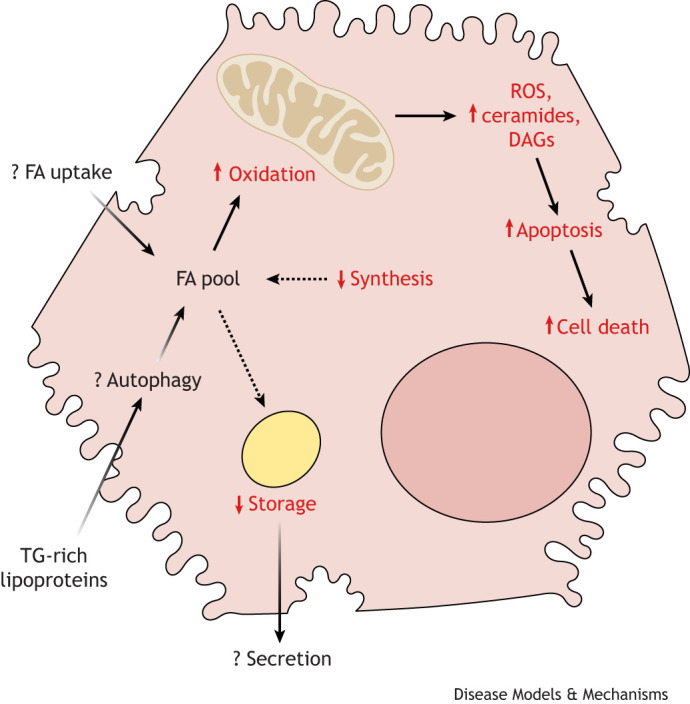
**Effects of FA composition on human hepatocellular lipid metabolism.** Schematic of a human hepatocyte cultured in palmitic acid (PA; a saturated FA) compared to hepatocytes cultured in oleic acid (OA; a monosaturated FA). Hepatocytes cultured in PA generally have higher levels of mitochondrial oxidation, which might increase reactive oxygen species (ROS) production, and that of ceramides and diacylglycerol (DAG), and also apoptosis. Hepatocytes cultured in PA have lower FA synthesis and TG storage compared to cells cultured in OA. ‘↑’, higher when cultured in PA than when cultured in OA; ‘↓’, lower when cultured in PA than when cultured in OA.

In human hepatocellular cancer cell lines [Huh7 (Box 1) and HepG2 (Box 1) cell lines] and primary human hepatocytes (PHHs; Box 1), the addition of FAs to the culture media reliably increases intrahepatocellular lipid content, whereas sugars appear to only increase lipid content in PHHs and to a lesser extent than FAs (Huggett et al., 2022). This might be because human hepatocellular cancer cells are highly proliferative and therefore more glycolytic than PHHs. Indeed, intrahepatocellular lipid content reportedly increases in these cell lines at higher, supraphysiological concentrations of glucose (Green et al., 2015a). Most *in vitro* models of hepatocellular TG accumulation rely on culturing cells in medium that is supplemented with a single FA [typically oleic acid (OA), a MUFA], or palmitic acid (PA; a SFA), for an acute period of time (between 8 and 48 h), which generally results in a dose-dependent increase in intracellular lipid accumulation, as measured by fluorescence or Oil Red O staining (as reviewed in Green et al., 2015b). Few studies provide a rationale for the concentration or duration of FA or sugar treatment that has been used in the *in vitro* cell studies, and which might not recapitulate the conditions driving *in vivo* human IHTG accumulation. Such short treatment periods might also model an acute insult to hepatocytes rather than recapitulate the chronic accumulation of intrahepatocellular TG, which is more representative of MASLD pathogenesis. Similarly, the use of a single FA is not representative of the FA mixture that hepatocytes are exposed to *in vivo* and does not consider the contribution of remnant TRLs, which also represent a major, but often overlooked, route of hepatic lipid delivery. Although there are relatively few *in vitro* studies that have investigated the effects of FA composition on hepatocellular fat metabolism (summarized in Fig. 3), they have provided some insights, which we discuss below, into the mechanisms that might underlie observations made in human *in vivo* studies.

### Effects of FA composition on intrahepatocellular TG storage

Cells cultured with PA have been shown to be less steatogenic than those cultured with OA (de Sousa et al., 2021; Eynaudi et al., 2021; Gómez-Lechón et al., 2007; Lee et al., 2010; Listenberger et al., 2003; Ricchi et al., 2009) because PA activates pro-apoptotic rather than storage pathways, rapidly leading to cell death (Listenberger et al., 2003). This PA-induced lipotoxicity (Box 1) can be rescued by the addition of OA, as OA promotes the storage of FAs as TG in LDs, which is less toxic to cells (Gómez-Lechón et al., 2007; Moravcová et al., 2015). Studies have also shown that cells cultured in different ratios of OA and PA show no significant differences in intracellular TG accumulation between the different OA:PA ratios (Gómez-Lechón et al., 2007; Moravcová et al., 2015; Nagarajan et al., 2022). These findings indicate that it is the lipotoxic effect of PA alone that causes the decreased intracellular accumulation of TG in cells. Studies that have directly compared the effects of PA and linoleic acid (LA; a PUFA) in HepG2 cells have demonstrated that LA is more steatogenic than PA (Teixeira et al., 2023), and that cells cultured in LA more rapidly accumulate fat than those cultured in PA (Paramitha et al., 2021).

These *in vitro* findings are in contrast to *in vivo* human studies, which show that consuming excess SFA compared to PUFA leads to more IHTG accumulation (Bjermo et al., 2012; Luukkonen et al., 2018; Rosqvist et al., 2014, 2019), highlighting the limitations of these cellular models in recapitulating the complex and dynamic nature of *in vivo* lipid flux to and from the liver. The limitations of these cellular models include that they do not undergo the regular transition from the fed to fasted state, nor any associated changes in pancreatic and gut hormones. They also do not mirror the two-dimensional *in vivo* environment in which adjacent cells, paracrine signals and the presence of blood fluid flow can modify hepatic FA metabolism. The genotype of the cell lines used for these studies might also have a large and often overlooked effect on intrahepatocellular TG storage. For example, HepG2 cells have been reported to carry the *PNPLA3* variant that is associated with PNPLA3 NAFLD and so might not be appropriate to use when modelling metabolic NAFLD (Green et al., 2015a; Luukkonen et al., 2016). Nevertheless, these models do provide unique insights into how FA composition might affect LD morphology and distribution. For example, confocal imaging and Raman spectroscopy studies have shown that cells treated with unsaturated FAs have larger LDs than those treated with SFAs, and, although the underlying cause of this observation remains unknown, it might be due to differences in the partitioning of FA into oxidation pathways (Eynaudi et al., 2021; Paramitha et al., 2021).

### Effects of FA composition on intrahepatocellular FA oxidation

In contrast to *in vivo* human studies, few *in vitro* studies have directly measured the partitioning of OA and PA towards oxidation pathways in cultured hepatocytes. However, it has been shown that HepG2 cells and primary rat hepatocytes treated with PA have higher levels of ceramide and DAG accumulation than hepatocytes treated with OA (Chabowski et al., 2013; Lee et al., 2010), indicating that the PA may be preferentially partitioning towards ceramide- and DAG-synthesis pathways rather than towards oxidation and storage pathways, compared to OA. In line with *in vivo* human studies, the accumulation of DAGs is associated with insulin resistance (Lee et al., 2010), but the role of DAGs in intrahepatocellular TG accumulation remains unclear.

Eynaudi et al. (2021) investigated the effects on LD–mitochondria interactions in HepG2 cells treated with PA or OA. In OA-treated HepG2 cells, larger LDs formed that were located closer to mitochondria than were the smaller LDs that formed in PA-treated cells. This difference in the size and location of LDs in OA- and PA-treated cells might be mediated by the differential expression of perilipin (PLIN) proteins (Box 1) which coat the LD surface and are involved in modulating LD morphology, biogenesis and FA metabolism (Chandrasekaran et al., 2024), as well as that of fusion/fission proteins on mitochondria. This is because PLIN5 and mitofusin-2 (MFN2) protein expression is observed on the LDs of OA-treated HepG2 cells, while PLIN2 and dynamin-related protein 1 (DRP1; also known as DNM1L) expression is observed on the mitochondria of PA-treated HepG2 cells (de Sousa et al., 2021; Eynaudi et al., 2021). Measurements of cellular adenosine triphosphate (ATP) concentration and of mitochondrial transmembrane potential indicate that OA-treated cells utilize mitochondrial ATP for TG production and storage, while LDs from PA-treated cells provide TG for mitochondrial FA oxidation (Eynaudi et al., 2021). These results are in line with those of Nakamura et al. (2009), who observed the increased expression of CPT-1, the rate-limiting enzyme in mitochondrial FA oxidation, in PA-treated compared to OA-treated cells. Nakamura et al. (2009) also demonstrated that PA induced the overproduction of reactive oxygen species (ROS; Box 1), which could be rescued by mitochondrial inhibitors but not by inhibitors of other parts of the ROS production pathway. These observations suggest that PA-induced ROS production *in vitro* in hepatocytes is mediated by an increase in mitochondrial oxidation. Taken together, these results indicate that PA-treated cells have a higher rate of FA oxidation than do OA-treated cells, which increases ROS production, mitochondrial dysfunction and lipotoxicity, and which might explain the higher levels of apoptosis reported in cells cultured in only PA.

However, comparisons between OA and PA in isolation might be misleading. When compared to LA-treated cells, PA-treated cells have lower levels of ROS production, suggesting that PUFAs, such as LA, promote even higher rates of FA oxidation than do SFAs or MUFAs. Indeed, by using stable isotope-labelled FA tracers and measuring media acetate, it has been shown that cells treated with a SFA- compared to a PUFA-enriched FA mixture have lower rates of β-oxidation (Nagarajan et al., 2022). These observations of Huh7 cells being treated with a physiological mixture of FAs (as opposed to a single FA in isolation) are in line with findings from *in vivo* human studies, which have shown that the oxidation of dietary SFAs occurs to a lesser extent than that of dietary PUFAs (Parry et al., 2021). This suggests that culturing hepatocytes in FA mixtures that mirror physiological FA compositions provides a better model of *in vivo* human FA metabolism than culturing with a single FA. It remains unclear how other relevant processes in FA metabolism, such as synthesis and esterification, are influenced by FA composition.

### FA composition and intrahepatocellular TG synthesis and breakdown

Several studies have shown that the transcriptional activity of sterol regulatory element-binding protein 1 [SREBP-1 (also known as SREBF1); Box 1], a key lipogenic transcription factor regulating DNL, was higher in cells cultured in OA than in those cultured in PA. This suggests that OA-induced steatosis might be partially driven by an increase in DNL (Lee et al., 2010; Ricchi et al., 2009). However, when cells cultured in SFA-enriched versus PUFA-enriched FA mixtures are compared, no differences in intrahepatocellular DNL-derived TG have been found between them, as measured by stable isotope tracers (Nagarajan et al., 2022). This lack of difference is in line with findings from human interventional studies (Luukkonen et al., 2018) but is in contrast to the observations from cell studies that compared the effects of a single FA on the contribution of DNL to hepatocellular TG (Lee et al., 2010; Ricchi et al., 2009). The discrepancy in findings from different cellular studies could, in part, be attributed to how intrahepatocellular DNL was assessed, with some measuring DNL by stable isotope tracers (Nagarajan et al., 2022) and others measuring SREBP-1 activation (Lee et al., 2010; Ricchi et al., 2009) along the duration cells were exposed to the FA.

Although few studies have examined the effect of FA composition on cytosolic lipolysis, there has been some interest in the effects of FA composition on lipophagy (Box 1). Autophagy (Box 1) is the degradation of intracellular cargo, including LDs (known as lipophagy), and it might have a quantitively important impact on IHTG turnover and accumulation (Singh et al., 2009). Liver biopsies from patients with NAFLD have shown an aberrant decrease in protein markers of autophagy compared to those without NAFLD, which might influence IHTG accumulation in these patients (Fukuo et al., 2014). SFA and unsaturated FAs might also have differential effects on autophagy, but the use of different cell lines and techniques to measure autophagic flux has provided conflicting results (Mei et al., 2011; Tu et al., 2014). As dietary FAs delivered to the liver in TG-rich TRLs can be liberated by autophagy, further work is needed to elucidate the role of autophagy on hepatic TG accumulation (Hornick et al., 1984). This route of TG delivery to intrahepatic FA pools is understudied as a potential mechanism for hepatic TG accumulation, as the majority of the focus to date has been on the contribution of non-esterified FA, which models FAs derived predominantly from adipose tissue. Although intracellular processes may, to a degree, influence intracellular FA composition, its major determinant is what hepatocytes are exposed to, which *in vivo* is mostly determined by dietary fat composition (Hodson et al., 2008).

### FA composition, hepatocellular FA uptake and TG secretion

Although hepatic FA uptake and VLDL secretion might play pivotal roles in IHTG accumulation, few studies have investigated the effects of FA composition on these processes. In primary rat hepatocytes treated with OA or PA, FA transporter protein expression was found to increase to a similar level as that in cells cultured in FA-free media (Chabowski et al., 2013). Similarly, our group has shown that human Huh7 cells cultured in SFA- or PUFA-enriched FA mixtures take up the same proportion of FAs in the media and secrete the same amount of TG (Nagarajan et al., 2022). However, it has also been shown in AML12 murine hepatocytes that PA treatment activates the mTORC1–IRE1α pathway and leads to higher levels of TG secretion than those observed in OA-treated cells (Wang et al., 2020). It is possible that the different cell lines used by these studies account for the discrepancies in these results, as different hepatocyte lineages are known to secrete different amounts and types of TG particles (Green et al., 2015b). Although few studies have investigated the effects of FA composition on VLDL clearance, composition and handling, differences in these processes might play a role in the divergent effects of dietary FA composition on human IHTG accumulation.

## Conclusions

Obesity is a risk factor for IHTG accumulation, and it is well documented that lifestyle choices, such as diet, can influence the risk of obesity and IHTG accumulation. Current evidence from hypercaloric feeding studies clearly shows that the consumption of excess calories, regardless of the macronutrient composition, increases IHTG content. What is emerging from dietary hypercaloric and isocaloric intervention studies in humans is that an individual's dietary fat composition likely plays a role in mediating their IHTG content and composition. Although the effect of SFA on IHTG content appears to diverge from that of unsaturated FA, specifically PUFA, the mechanisms underpinning these observations remain to be elucidated. This is in part due to the challenging nature of studying *in vivo* human liver fat metabolism. Although advances in imaging technologies now allow researchers to quantify and assess IHTG content and composition, we know relatively little about the location and morphology of the LDs that make up the IHTG content. This is where there is a need for cellular models that can recapitulate lipid accumulation (including LD morphology and composition), along with *in vivo* human liver fat metabolism (including, DNL, FA oxidation, secretion of VLDL size particles, synthesis of ceramides, DAGs and inflammation, etc.), by utilizing mixtures of FAs and sugars at physiological concentrations and ratios, and culture durations that enable researchers to investigate the more chronic, rather than acute, responses. The use of functional metabolic assessments (such as measuring DNL or FA oxidation using isotopic tracers either in a basal state or in response to physiological stressors like a bolus of nutrients/substrates or rapid elevation of hormones), in combination with gene and/or protein expression measurements, also provides a deeper insight into the pathways that might be involved in excessive hepatocellular TG accumulation and that could be targeted to lower IHTG accumulation and thus the risk of any associated metabolic disease.
